# The Nexus between Green Perceived Quality, Green Satisfaction, Green Trust, and Customers’ Green Behavioral Intentions in Eco-Friendly Hotels: A Structural Equation Modeling Approach

**DOI:** 10.3390/ijerph192316195

**Published:** 2022-12-03

**Authors:** Magdy El-Sayed Hashish, Ahmed Hassan Abdou, Shaimaa Abo Khangar Mohamed, Ahmed Saleh Abo Elenain, Wagih Salama

**Affiliations:** 1 Social Studies Department, College of Arts, King Faisal University, Al-Ahsa 31982, Saudi Arabia; 2 Department of History, College of Arts, Mansoura University, Mansoura 35516, Egypt; 3 Hotel Studies Department, Faculty of Tourism and Hotels, Mansoura University, Mansoura 35516, Egypt; 4 Tourism Studies Department, Faculty of Tourism and Hotels, Mansoura University, Mansoura 35516, Egypt; 5 Department of Hotel Studies, High Institute of Tourism and Hotels, Ismailia 41511, Egypt

**Keywords:** green perceived quality, green satisfaction, green trust, eco-friendly, green behavioral intentions

## Abstract

With the growing awareness of the climate change effects, hotel customers progressively intend to favor green products and services that minimize adverse environmental effects. The key factors affecting customers’ green behavioral intentions in the hospitality industry context are still under research. Accordingly, this study primarily aims at empirically investigating the nexus between green perceived quality (GPQ), green satisfaction (GS), green trust (GT), and customers’ green behavioral intentions (CGBIs) in a sample of five-star eco-friendly hotels in Egypt. More specifically, the study first endeavors to investigate the direct impact of GPQ on GS, GT, and CGBIs besides exploring the direct impact of GS and GT on CGBIs in addition to examining the potential effect of GS and GT as mediators in the nexus between GPQ and CGBIs. To achieve the study’s aim and associated objectives, a self-administrated questionnaire was developed and distributed to a convenience sample of local guests staying at certified five-green star hotels. A total of 500 questionnaires were distributed, and only 304 valid forms, representing 60.8%, were used in the statistical analysis. Seven hypotheses, reflecting the direct and indirect relationships between study constructs, were examined by using Structural Equation Modeling (SEM) with bootstrapping technique. The study findings revealed that GS, GT as well as CGBIs are significantly positively affected by GPQ, respectively. Furthermore, GT and GS have a significant positive effect on CGBIs. Moreover, GT as well as GS partially mediate the nexus between GPQ and CGBIs. From the previous findings, it could be concluded that the increase in investment in enhancing GPQ significantly contributes to the improvement in GS, GT, and CGBIs. Additionally, the higher the GT, GPQ, and GS, the greater the revisit intention to green hotels, positive green word-of-mouth (GWoM), and intention to pay a premium for staying in environmentally friendly hotels. As a result, for enhancing CGBIs and sustaining a customer-hotel long-term relationship, hotel operators should make efforts towards maintaining GT, improving GPQ, and increasing customers’ GS as key predictors of CGBIs in the hotel industry context.

## 1. Introduction

With the increased awareness of climate change impacts, customers are progressively preferring to purchase goods and services that support environmental/green initiatives and strive to minimize adverse environmental impacts [1]. Globally, companies are encountering the need to incorporate environmental friendliness into their strategies, policies, and practices owing to the growing pressures in terms of environmentally sustainable development and climatic change [2]. In the recent years, both customers and policymakers have become extremely concerned about the detrimental effects caused by the hospitality industry on the environment [3]. The hotel industry is under pressure from its customers to adopt environmentally friendly practices that meet their needs and expectations [4]. Customers progressively look for staying at environmentally friendly hotels with minimal ecological impacts [5]. The hotel industry is growing considering this environmental dimension in its quest to deliver positive and satisfying experiences to its customers [6]. The collective customers’ awareness of environmental sustainability and concern about the environment have greatly contributed to the increased demand for green hospitality products such as eco-friendly/green hotels, green restaurants, green café, green cruises, and green resorts [7,8,9,10,11]. For example, the empirical study conducted by Virginia Polytechnic Institute and State University on a sample of 489 air travelers aimed at identifying their perceptions towards environmentally friendly hotels revealed that only 3% of the respondents had a negative perception about the eco-friendly hotels, and 70% stated that they prefer to choose an environmentally friendly hotel over an ordinary one [12]. Moreover, 78% of the respondents in Miller’s study [13] found that they either always or sometimes try to find environmental information about the destination they intend to visit. 

A substantial amount of attention has been given to green consumer behavioral intentions in the hotel literature in the last few years [14,15,16,17,18]. A customer’s behavioral intention (CBI) reflects his or her likelihood of engaging in a specific behavior [19]. Consumer adoption intentions for green hotels have been explored extensively, specifically those related to willingness to stay in eco-friendly hotels [17], willingness to pay a premium [20], and intentions to positive word-of-mouth [21]. Based on the theory of planned behavior (TPB), several empirical and theoretical studies have explored consumers’ behavioral intentions toward green hotels or restaurants based on particular norms, attitudes, and perceived behavioral controls, i.e., [22,23,24,25,26]. Furthermore, more and more green determinants have been employed for identifying their impacts on CGBIs. Some of these determinants include the green perceived quality (GPQ), green satisfaction (GS), and green trust (GT). 

GPQ has been described as the judgment of the customer about a product’s/brand’s overall environmental superiority or excellence [1]. Numerous studies have explored the significant positive role of GPQ in predicting CGBIs, such as green purchase intention (GPI), green word-of-mouth (GWoM) as well as the willingness and acceptance to pay more to stay at green/eco-friendly hotels [25,27,28,29,30]. Furthermore, GS has been characterized as a pleasing consumption-related fulfillment level that meets customers’ sustainable expectations and satisfies their environmental needs, desires, and green demands [31]. In the context of green marketing, GS is the key predictor of CGBIs. Various scholars have revealed that GS significantly positively influences GWoM [32], green customer loyalty [22] as well as purchase intention [33]. Gao et al. [28], in their meta-analysis, revealed that perceptions of the firm (i.e., GPQ, and GS) have a great positive effect (*r* = 0.424) on CBI toward green hotels/restaurants (i.e., revisit, repurchase, retention, WoM intentions, intention to pay a premium, and willingness to pay). Chen [31] p. 312 defined GT as “*a willingness to depend on a product, service, or brand based on the belief or expectation resulting from its credibility, benevolence, and ability about its environmental performance*”. As a key factor in sustaining long-term customer relationships, GT has a significant influence on customers’ purchase and repurchase intentions, intent to revisit, and willingness to pay a premium for green products and services [34,35,36,37]. 

Although several studies have explored the interrelationships among GPQ, GS, GT, and CGBIs in diverse contexts, the nexus between these constructs in the green hotel industry context is still inadequate, particularly in the developing countries. According to Myung et al. [38], most of the environmental-related research in the hospitality industry context was undertaken in the developed countries, while environmental-related research focusing on developing countries is still inadequate. Furthermore, to the best of knowledge of the authors of this study, the mediating effect of GT and GS in the nexus between GPQ and CGBIs in the green/eco-friendly hotels context has not been examined before. Moreover, Gao et al. [28], through their meta-analysis, found that more than 60% of the articles included in their study (26 articles) utilized the theory of planned behavior or the theory of reasoned action as a theoretical lens to explain consumer green behavior intentions. To make imperative theoretical contributions, they recommended hospitality academicians and researchers to improve existing models or seek out novel and more diverse theoretical frameworks. As a result, for fulfilling the gap in green marketing literature regarding the hospitality sector, the present study primarily aims to empirically investigate the nexuses between green perceived quality (GPQ), green satisfaction (GS), green trust (GT), and customers’ green behavioral intentions (CGBIs) in a sample of five-star eco-friendly hotels in Egypt. More specifically, the study seeks to investigate the direct impact of GPQ on GS, GT, and CGBIs besides exploring the direct impact of GS and GT on CGBIs. In addition, based on the Stimulus-Organism-Response (S-O-R) model, the study seeks to examine the potential mediating effect of GT and GS on the nexus between GPQ and CGBIs. To achieve the study’s aim and associated objectives, a self-administrated questionnaire will be developed and distributed to a sample of guests staying at certified five-green star hotels, who will be asked to answer the following questions: (1) How does the GPQ directly affect GS, GT, and CGBIs? (2) How do GS and GT directly affect CGBIs? (3) What is the potential mediating effect of GT and GS on the nexus between GPQ, and CGBIs? 

Compared to the previous literature on CGBIs, the possible contributions of this study are as follows. Firstly, a novel research framework was developed and tested where GPQ, GS, and GT act as determinants of CGBIs in the green/eco-friendly hotel context. These findings may provide hotel operators, seeking for achieving competitive advantages in the green hotel industry context, with an in-depth understanding of the key predictors that significantly affect CGBIs which should be considered in their strategic plans. Secondly, in response to calls from Gao et al. [28] to utilize a new theoretical framework to explore the antecedents of the CGBIs, the study will use a Stimulus-Organism-Response (S-O-R) model to examine the potential effect of GT and GS as intermediary variables in the GPQ-CGBIs relationship. This may be the first endeavor to use this model in predicting CGBIs indirectly through GS and GT in the hotel industry context. Thirdly, the study explores the role of the previous constructs in predicting CGBIs in the developing countries, where the GPQ, GS, GT as well as CGBIs may differ from those in developed ones. Finally, the developed model may serve as a basis for hospitality scholars’ forthcoming research examining GPQ, GT, GS, and CGBIs in diverse hospitality sectors.

The study is structured as follows. Following the introduction, Section 2 focuses on reviewing the literature and developing hypotheses regarding the nexus between GPQ, GS, GT, and CGBIs. Data collection, measures and instruments, samples, and the statistical methods used for data analysis have been described in Section 3. Furthermore, Section 4 deliberates on descriptive statistics, confirmatory factor analysis, reliability and validity of measures, and structural equation modeling (SEM) with bootstrapping. The study findings are discussed in Section 5. Finally, Section 6 (conclusion of the study) summarizes the theoretical and practical implications along with the limitations of the current study and areas of future research.

## 2. Theoretical Background and Hypotheses Development

The earth’s ecosystem has been greatly affected by human activities for thousands of years. Recently, the detrimental impacts of human practices and actions could be seen everywhere [12]. As one of the largest industries in the world, the hospitality industry contributes significantly to these issues [39]. In the tourism and hospitality industry context, hotels are profoundly water- and energy-intensive sectors. Owing to their huge consumption of energy and resources (such as lighting, water, and many disposable products) on daily basis, hotels have posed a significant environmental challenge [40,41]. Recently, the public has become more concerned about the environmental issues, and consumers have become more environmentally aware than they were in the past [3]. Customers are more and more favoring green consumption as an effective method of protecting the environment [42]. Taking into consideration these phenomena, a growing number of hotels are adopting green practices and implementing environmental programs, resulting in so-called eco-friendly hotels, to increase their market share besides achieving a competitive advantage over their peers [43,44,45]. 

In the hospitality industry, eco-friendly hotels are time and again denoted by several alternative terms. Some of these terms are sustainable hotels, green hotels, environmentally responsible hotels, and environment-friendly hotels [11,19,20,21,22,23,46]. Green or eco-friendly hotels were defined by the green hotel association [47] p. 1 as “*environmentally friendly properties whose managers are eager to introduce programs that save water and energy, and reduce solid waste—while, at the same time, saving money—to help protect both the earth and the environment"*. In accordance with Chen [48], the implementation of an environmental management system that meets international standards is an imperative component of becoming an eco-friendly hotel. This system must be applied to all spheres of the hotel along with guests, suppliers, and local communities. Others referred to eco-friendly hotels as environmentally friendly properties that adopt various ecological practices that are associated with water conservation, energy saving, and waste reduction [49]. 

A shift in consumers’ attitudes and behavior regarding sustainable products and services, particularly in the hospitality industry context, has occurred over the last three decades [50]. Tourists’ environmental interest has steadily increased more than it was in the past. Numerous scholars have studied the perceptions of customers toward green/eco-friendly hotels and their impacts on intentions to green hotels. For instance, an empirical investigation conducted by Hou and Wu [16] revealed that tourists’ intention of staying in green hotels is significantly influenced by the level of their awareness and perceptions of green building design attributes. In the Jordanian green hotel context, a positive but moderate correlation has been found between tourists’ perceptions of environmental practices and their stay in eco-hotels [51]. A relevant study explored the influence of consumers’ environmental awareness on their intention to visit green hotels in north Cyprus, and found that environmental awareness and concern directly and positively influenced the guests’ intentions to visit hotels [52]. Abdou et al. [7] explored the effect of perceived environmentally sustainable practices (ESPs) on customer citizenship behavior (CCB) in eco-friendly hotels, and illustrated that ESPs significantly contribute to enhancing CCBs. Another empirical study conducted by Martínez García de Leaniz et al. [22] revealed that tourists’ intention to stay at certified environmentally responsible hotels and intention to spread positive WOM are significantly influenced by tourists’ environmental consciousnesses.

In addition to the previous studies, numerous studies have examined other determinants affecting CGBIs in the tourism and hospitality industry context. For example, in their meta-analysis, Gao et al. [28] classified these determinants into two categories, namely internalized perceptions (i.e., personal value, attitude, perceived benefits, and environmental knowledge and awareness) and perception of the firm which included firm image, perceived quality as well as customer satisfaction. In this study, we will primarily focus on three key antecedents of CGBIs, namely GPQ, GS, and GT.

In response to Gao et al. [28] the theoretical framework of this study is grounded on the Stimulus-Organism-Response (S-O-R) model to explore customer green behavior intentions in eco-friendly hotels. T.M. et al. [53], in a recent systematic literature review aiming to identify and analyze 76 studies addressing the consumer adoption of green hotels, suggested that SOR is an ideal framework that effectively encompasses several antecedents, moderators, mediators, and outcomes. In the S-O-R framework, the behavioral outcomes are determined by three components, namely stimulus, organism, and response. This model, which was initially proposed by Mehrabian and Russell in 1974, advocates that environmental stimuli affect a person’s cognitive and affective reactions, resulting in response behavior. The stimuli (S) are referred to as “the influence on the individual,” and are the external factors that affect the mental state of an individual. These external factors include different forces of the physical environment [54]. In this model, an organism (O) is the internal structure and process between a person’s external stimuli and his/her final reaction, action, or response (R). In the intervention process, a variety of perception, physiological, sensory, and thinking activities are involved [55]. In response to external stimuli, organisms’ internal states are impacted by these factors, further influencing their behavior. People’s response consists of their mental responses or their behavior, which may be verbal or non-verbal, an avoidance response, or an approach response [56]. In other words, significant environmental changes can affect an individual’s psychological and emotional stability, further influencing their behavior. As an underpinning theory, this model has been used to study how external stimuli affect customers’ green behavioral intentions. In this study, GPQ was employed as the stimulus (S), GS and GT were employed as organism-related factors (O), and CGBIs were used as a response variable (R). 

### 2.1. The Nexus between GPQ and CGBIs

In the previous studies, perceived quality (PQ) was cited as a vital factor in predicting customer behavior and sustaining long-term customer relationships, i.e., [29,30]. In the green marketing context, numerous studies have examined the nexus between GPQ and CGBIs in diverse contexts, and confirmed the positive significant impact of GPQ on CGBIs. For instance, in the green information technology context, a study conducted by Gil and Jacob [29], on a sample of 496 professionals, revealed that GPQ is positively and significantly associated with customers’ green purchase intention. Similarly, Cheung et al. [30] validated that GPQ directly influences GBI. Furthermore, in Pakistan, the recent empirical investigation conducted to explore the effect of GPQ on GPI among energy savers validated the direct positive and significant relationship between GPQ and GPI (*β* = 0.260, *p* < 0.05) [43]. Chen et al. [32] established that green word-of-mouth is significantly impacted by GPQ (*β* = 0.228, *p* < 0.05). In the hotel industry context, Alexandris et al. [57] concluded that perceived service quality has a very high proportion of variance in WoM communication and purchase intention (93%, and 85%, respectively) along with a modest proportion of variance in price sensitivity (21%) among hotel guests in north Greece. In the green restaurant context, green consumers are more likely to return to green restaurants if the perceived green quality (i.e., food quality, service quality, and ambiance quality) is high [58]. A significant role has been assigned to GPQ in the nexus between green consumerism and revisiting green restaurant intentions. Consumers will be more likely to revisit a restaurant if they perceive its food and service as of high quality [25]. Accordingly, it could be presumed that the higher GPQ in eco-friendly/green hotels would lead to higher CGBIs. Hence, the following hypothesis could be suggested:

**H_1_**: *GPQ has a significant positive effect on CGBIs in green/eco-friendly hotels*.

### 2.2. The Nexus between GPQ and GS

Many empirical studies have been undertaken in marketing fields to understand the influence of perceived quality on customer satisfaction, i.e., [1,59,60]. Perceived quality is an imperative determinant of customer satisfaction [14,61]. Previous research indicates that there is a positive association between perceived quality and customer satisfaction since the perceived quality of products and services significantly positively contributes to enhancing consumer satisfaction [15]. 

The tourism and hospitality literature lays emphasis on the significance of the association between service quality and customer satisfaction [62]. The previous studies established the relevance of this relationship for hotels’ and restaurants’ success [63,64]. For instance, in the yoga tourism context, Abdou et al. [59] revealed that perceived service quality has a significant positive impact on yoga tourists’ satisfaction. Furthermore, by using Gronroos’ service quality model, Zaibaf et al. [64] examined the effect of perceived quality on customer satisfaction in the hospitality industry context. Their findings proved that the increase in perceived service quality significantly positively enhances customer overall satisfaction. In the green marketing context, several empirical investigations reflect the influence of GPQ on GS. Imaningsih [60] found that GPQ has a significant positive impact on GS (*β* = 0.378, *t* = 3.082, *p* < 0.01). Furthermore, a relevant study conducted by Chen and Chang [1] on a sample of Taiwan’s consumers who have the purchase experience of information and electronics products concluded that GPQ significantly positively contributes to enhancing GS. Upon the previous findings, it could be postulated that the higher the GPQ, the better GS. Hence, we hypothesized that:

**H_2_**: *GPQ has a significant positive effect on GS in green/eco-friendly hotels.*

### 2.3. The Nexus between GPQ and GT

A company’s green trust is a measure of how confident consumers are that it performs well in terms of sustainability [1]. GPQ is considered to be one of the key predictors of GT. Several scholars clarified that GT is significantly affected by GPQ. For example, Chen and Chang [1], in their empirical study on a sample of Taiwanese customers, specified that GPQ positively and significantly impacts GT. Furthermore, Chen et al. [65] concluded that GT is significantly associated with GPQ (*β* = 0.663, *p* < 0.01). The findings of an empirical study on the field of information technology in India by Gil and Jacob [29] revealed that GT is directly enhanced by GPQ (*β* = 0.40, *t* = 9.71, *p* < 0.01). In addition, Cecillia and Tanamal [66], in their study on a sample of 65 Apple consumers in Surabaya, clarified that GPQ is one of the key antecedents of GT. Another study conducted by Sabono and Murwaningsari [67] on Indonesian consumers suggested that GT is significantly influenced by GPQ. They stated that in order to trust a product, the customer must feel the best quality of the product. In Surabaya, Yuwono [68] revealed that GPQ has a significant positive impact on GT (*β* = 0.23, *t* = 2.76, *p* < 0.05). Given the above, we expected that the GPQ would enhance the customers’ GT. Hence, we hypothesized that:

**H_3_**: *GPQ has a significant positive effect on GT in green/eco-friendly hotels.*

### 2.4. The Nexus between GS and CGBIs

From a psychological point of view, satisfaction is the feeling of pleasure one experiences after receiving an attractive product or service that meets his/her expectations [69]. Several researchers have illustrated that GS plays a significant role in predicting CGBIs in diverse contexts. For example, in the context of green information and electronics products, GS is positively and significantly associated with GWoM [32]. In Jordan’s Fast-moving consumer goods (FMCG) industry, Al- Quran et al. [70] further verified that GS has a positive significant relationship with GPI. Lam et al. [71] established that customer satisfaction has a significant positive impact on customer repurchase intention of green products (*β* = 0.18, *t* = 2.87, *p* < 0.01). Similarly, the previous study conducted by Ranaweera and Prabhu [33] revealed that customer satisfaction significantly influences not only customer retention but also future purchases. Martínez et al. [22] suggested that green customer loyalty, in the Spanish hotel industry context, is significantly increased by GS (*β* = 0.251, *p* < 0.05). Likewise, in the context of the Chinese green hotel industry, Wang et al. [21] specified that customers’ WoM intention has been impacted by their GS (*β* = 0.73, *p* < 0.001). Hence, the following hypothesis is proposed:

**H_4_**: *GS has a significant positive effect on CGBIs in green/eco-friendly hotels*.

### 2.5. The Nexus between GT and CGBIs

The previous studies that have been built based on attitude-behavior relationships revealed that GT in the product/brand can subsequently enhance CGBIs such as GPI, GPI, GWoM intentions, premium purchase intentions, and loyalty behavioral intentions [36,37,72,73,74]. Research on green marketing has revealed the significance of GT in predicting GPIs [34,73]. In the era of the COVID-19 pandemic, the findings of the empirical study conducted by Jian et al. [36], aimed to observe the impact of green hotel brand trust (GHBT) on CBIs, concluded that GHBT is positively and significantly correlated to the intention to pay the premium (*β* = 0.73, *t* = 18.760, *p* < 0.001) as well as the intention to make sacrifices for staying at an environmentally friendly hotel (*β* = 0.12, *t* = 2.543, *p* < 0.01). Konuk et al. [37], in their cross-country study, revealed that GT is significantly and positively associated with WoM intention, GPI, and intention to pay more for green products. Wasaya et al. [72] advocated that GPI among energy savers in Pakistan is significantly affected by GT (*β* = 0.281, *t* = 4.044, *p* < 0.001). In the American hotel context, GT significantly promotes customers’ revisit intention and intention to participate in the green practices, and had a negative impact on negative word of mouth (NWoM) intention [35]. Likewise, in the Spanish hotel industry context, Martínez et al. [22] suggested that customers’ green loyalty is highly significantly affected by GT (*β* = 0.593, *p* < 0.05). Given the above, we predicted that CGBIs would be positively and significantly affected by the high level of GT in green hotel products and services and vice versa. Accordingly, we hypothesized that:

**H_5_**: *GT has a significant positive effect on CGBIs in green/eco-friendly hotels*.

### 2.6. The Mediating Role of GS in the Nexus between GPQ and CGBIs

Although numerous studies have investigated the direct relationship between GPQ and CGBI, limited empirical investigations have explored the role of GS as a mediator in the nexus between GPQ and CGBIs. An empirical investigation conducted by Bou-Llusar et al. [61] specified that customer satisfaction partially mediates the association between firm-perceived quality and customer purchase intention. Furthermore, in the organic food restaurant setting, Konuk et al. [37] examined the mediation effect of customer satisfaction in perceived food quality-revisit intention and perceived food quality-WoM intention relationships. The findings of the study concluded that customer satisfaction plays a significant partial mediating role in both relationships. Assaker et al. [14], in their empirical investigation of the UK upscale hotel context, confirmed that customer satisfaction has a full mediation effect on the hotel-perceived quality- guests’ loyalty (behavioral) intention relationship. Based on Stimulus-Organism-Response (S-O-R) model, green perceived quality (stimulus) could positively influence green satisfaction (organism), which significantly contributes to improving CGBIs (response). Upon the previous results concerning the direct and indirect relationship between GPQ and CGBIs, the extant literature indicates that while the causal relationship between GPQ and GS has existed, GPQ is generally referred to as a significant predictor of GS. Furthermore, numerous scholars clarified that GS significantly positively enhances CGBIs (i.e., green loyalty intention, green WoM, green purchasing intentions, willingness to pay more etc.). As a result, we suggested that:

**H_6_**: *GS has a significant mediating effect on the nexus between GPQ and CGBIs in green/eco-friendly hotels.*

### 2.7. The Mediating Role of GT in the Nexus between GPQ, and CGBIs

The mediating role of GT in the nexus between GPQ and CGBIs is still limited, especially in the green hotel context. Limited studies have examined the role of GT as an intermediary variable in the GPQ-CGBIs relationship. For instance, Gil and Jacob [29] concluded that the nexus between GPQ and GPI is positively and significantly mediated by GT. Similarly, Sabono and Murwaningsari [67], in their empirical study, advocated that GT plays a partial mediating role in the nexus between GPQ and customers’ green repurchase intention. In the upscale hotel context, the results established that trust fully mediates the influence of hotel PQ on guests’ loyalty (behavioral) intentions [14]. In addition, in the Korean food restaurant context, trust partially mediates the indirect effect of perceived service quality on behavioral intention [75]. Finally, based on the Stimulus-Organism-Response (S-O-R) model, the perceptions of green quality (S) could significantly contribute to affecting green trust (O), which may significantly enhance CGBIs(R). As a result, we assumed that:

**H_7_**: *GT has a significant mediating effect on the nexus between GPQ and CGBIs in green/eco-friendly hotels.*

**Figure 1:**
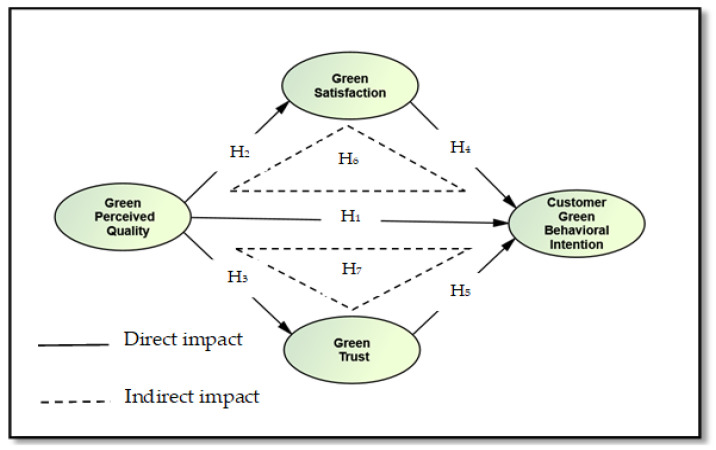
The proposed conceptual framework.

Figure 1 shows the proposed conceptual framework for this study. The framework treats GPQ, GS, and GT as the independent variables, whereas CGBI is regarded as the dependent variable. Based on the (S-O-R) model, GS and GT are also used as intermediary variables to examine the indirect relationship between GPO and CGBIs.

## 3. Materials and Methods

### 3.1. Measures and Instrument Development

To test the hypotheses explained in the conceptual framework, a cross-sectional survey method was used to empirically investigate the nexus between GPQ, GS, GT, and CGBIs. A self-administrated questionnaire was used to gather the data in this study. In order to develop the questionnaire, we reviewed a wide-ranging literature to determine frequently used and valid measures. Five sections were included in the questionnaire. Participants’ age, gender, educational level, monthly income, and marital status were listed in the first section. Section 2 focused on ascertaining how the investigated participants perceived green quality. GS as well as GT were examined in sections three and four, respectively. Customers’ green behavioral intentions were ascertained in the fifth section. 

For measuring GPQ among the investigated participants, a five-item scale proposed by Chen and Chang [1] was adapted and utilized. A sample item of this scale is “*the hotel’s products/services quality are durable in terms of environmental performance*”. The GPQ scale showed excellent internal consistency reliability (α = 0.959). Further, the green satisfaction scale utilized by Abdou et al. [76] was employed to ascertain the participants’ green satisfaction with hotel products and services. The scale is composed of four items. A sample of these items is “*Generally, you are pleased with the hotel’s eco-friendly products/services*”. The green satisfaction scale had good internal consistency reliability of 0.863.

A modified five-item Chen’s [31] scale was used to measure the green trust among the investigated participants. A sample of these items include “*In general, you think that claims made about the hotel’s environmental impacts are trustworthy*” and “*The hotel is keeping its promises regarding environmental improvement*”. Excellent internal consistency is demonstrated by this scale (α = 0.944). Finally, CGBIs were measured using a three-item measurement scale adopted by Martínez et al. [22]. These items reflect participants’ behavioral intentions toward staying at green/eco-friendly hotels, recommendations for environmentally friendly hotels, and intention to pay more for staying at green/eco-friendly hotels. The CGCB scale had an internal consistency of 0.927. All study items were rated on a 5-point Likert scale, with 1 equaling strongly disagree and 5 equaling strongly agree. In general, higher average scores indicate higher levels of GPQ, GS, GT, and CGBIs. All of the study’s constructs and their associated items are presented in Appendix A.

Firstly, the questionnaire form was developed in English and later translated into Arabic by two researchers fluent in both languages. To verify that there were no linguistic differences between the two versions after the Arabic translation had been completed, the survey was back-translated from Arabic to English by another two experts. No differences were found between the revised-translated version and the original English one. The survey questionnaire for this study necessitates high levels of content validity. To ensure that the content validity of the questionnaire is accurate, and it was developed to measure the variables that it is intended to measure, five hospitality scholars specialized in sustainability and environmental management were asked to review the survey content and give feedback. Moreover, 30 participants—who were not included in the main study sample—took part in a pilot study to validate the questionnaire’s clarity, simplicity, and consistency besides identifying any ambiguities between terms and meanings. Consequently, the wordings of some statements in the questionnaire were modified in the light of the feedback from participants and scholars. Furthermore, some statements were rearranged and reorganized.

### 3.2. Data Collection and Sample

As mentioned earlier, this study aims at empirically exploring the nexus between GPQ, GS, GT, and CGBIs in a sample of five-star eco-friendly hotels in Egypt. To fulfill the aim of the study, a self-administered questionnaire was developed and utilized for gathering data. The unit of analysis in this study is the customer level. Data was collected from local guests who have stayed in certified five-green star hotels. As mentioned by Abdou et al. [44], five-star eco-friendly hotels are more committed to implementing environmentally sustainable practices than any other category in Egyptian destinations. Hotels in the South Sinai Governorate, where most of the green star hotels and resorts are located [76], were invited to participate in the field study. Another reason is that approximately 90% of Egyptian tourism investment is concentrated in the coastal resorts/hotels of South Sinai, making it one of the fastest-growing tourist destinations in the world [77]. Among thirty-two certified five green star hotels in Egypt, as published on the green star hotel website [78], only ten hotels showed their willingness to participate in this study. 

With the permission of each hotel’s management, potential participants were asked to complete the questionnaire form during the check-out process. Research participants were selected using a nonprobability sampling technique (convenience sampling). From July–September 2022, 500 questionnaires were distributed, with only 50 forms distributed to each hotel. In total, the number of retrieved questionnaires was 348. For statistical analysis, only 304 forms (representing 60.8%) were found valid. 

Following Nunnally’s [79] criteria, the appropriate sample size was determined. According to his recommendations, the sample size should be calculated based on how many items will be examined. It is acceptable to maintain a ratio of 1 to 10 (item: sample). Thus, 170 participants were needed for the analysis of 17 items. This study consisted of 304 participants, which was an adequate sample size. Table 1 shows the demographic characteristics of the investigated respondents.

### 3.3. Data Analysis

The data analysis of this study was performed via SPSS version 22 and AMOS version 24. A descriptive statistical analysis was employed for analyzing the collected data; means, percentages, frequencies, and standard deviations provided an overview of the participants’ demographic data and their perceptions of the study constructs’ items. Study items’ reliability and validity were validated and evaluated using Cronbach’s alpha along with confirmatory factor analysis. To confirm convergence validity, composite reliability (CR) and the average variance extracted (AVE) were used. Additionally, the Fornell–Larcker criterion and the Heterotrait–Monotrait Ratio (HTMT) were used to evaluate discriminant validity. To detect common method variance (CMV), Harman’s single-factor test was employed. Finally, a Structural Equation Modeling (SEM) with bootstrapping was used to determine direct and indirect nexuses among the study constructs.

## 4. Results

### 4.1. Analysis of Demographic Characteristics of Participants

Based on the valid responses of 304 participants, Table 1 shows that more than half of the investigated participants (54.6%) were females, while 45.4% were males. Regarding the participants’ age, results indicate that those with an average age of 30 to 40 years constituted the largest percentage (36.2%) followed by those with an average age of 18 to 30 years (31.9%). As far as the educational level is concerned, the majority of the participants (60.9%) hold a university degree, followed by those holding postgraduate degrees such as master and doctorate degrees (28.6%). In regard to income, 37.8% earned USD 3001 to USD 4000 monthly. In terms of marital status, 54.6% of participants were single. 

### 4.2. Common Method Variance/Bias (CMV)

A common method variance/bias may exist since the data was collected by a self-administrated questionnaire. To reduce the probability of CMV, three approaches were employed: anonymity, confidentiality, and honesty [80]. The researchers informed participants that their information and responses would remain confidential and anonymous, and will be used only for research purposes. Response bias is less likely to be detected when anonymity is assured [81]. In addition, the participants were encouraged to answer all questions honestly, without considering any answer as false or true. Response bias decreases as honesty is confirmed [82]. In addition, Harman’s single-factor test was employed for the detection of CMV. In accordance with the findings of the exploratory factor analysis, 36.01% of the variance can be explained by one factor. CMV may be an issue when one factor explains the majority of variance and exceeds the threshold value of 50%. As a result, CMV did not pose a significant problem for this study [83].

### 4.3. Descriptive Statistics

The mean and standard deviation of the examined constructs and related items are presented in Table 2. Participants rated the GPQ at a higher level with an average mean in the range of 4.31 to 4.48. They strongly perceived that the hotel’s products/services quality is reliable in terms of environmental requirements and durability regarding the environmental performance. In terms of GS, participants were generally highly satisfied with purchasing the hotel’s products/services since they were environmentally friendly (M = 4.35, S.D. = 0.826). Regarding the GT, participants strongly perceived that the hotel’s environmental image is reliable, and the hotel’s environmental functionality is generally dependable with an average mean of 4.35 and 4.30, respectively. Regarding customer green behavioral intentions, the investigated participants really intended to stay in and positively recommend certified green hotels with mean ratings of 4.22 and 4.21, respectively.

### 4.4. Measurement Model

Using the maximum likelihood estimation method, CFA was conducted to ascertain the reliability and validity of the study constructs. Results shown in Table 2 revealed that composite reliability (CR) as well as Cronbach’s alpha values for all latent variables surpassed the recommended 0.80 thresholds [84], indicating acceptable internal reliability with CR values ranging from 0.874 to 0. 949 and Cronbach’s alpha values ranging from 0.863 to 0.959.

To evaluate the study construct validity, convergent and discriminant validities were studied. A factor loading of 0.50 and an average variance extracted (AVE) coefficient above 0.50 are necessary to achieve converging validity [85]. All study items loaded above 0.50, with a significant p-value (*p* < 0.001), and each construct’s AVE score ranged from 0.639 to 0.825, indicating that convergent validity has been achieved. Additionally, to verify the measurement model’s discriminant validity, two statistical pieces of evidence were used. Taking into consideration Fornell–Larcker’s criterion to maintain discriminant validity, the square root of AVE of every construct must be greater than its correlation with another construct. As shown in Table 3, all constructs’ AVE square roots (the diagonal numbers) are greater than their correlations with other constructs. Furthermore, another approach (HTMT) was employed. Henseler et al. [86] confirmed that when the HTMT value reaches 0.85 or higher, discriminant validity is compromised. As shown in Table 4, HTMT values for all latent construct pairs are below 0.85, indicating discriminant validity.

Several goodness-of-fit criteria were utilized to assess the fit of the measurement model. The values of “Root-Mean Square Error of Approximation (RMSEA)” and “Root Mean Square Residual (RMR)” were lower than 0.08 at 0.075 and 0.051, respectively. Furthermore, the “normed chi-square” (x^2^/df) value was lower than 5 at 3.179. Additionally, the values of “Comparative Fit Index (CFI)”, “Normed Fit Index (NFI)”, “Goodness of Fit Index (GFI)”, “Relative Fit Index (RFI)” and “Incremental Fit Index (IFI)” surpassed the cut-off value of 0.90 as recommended by Hair et al. [85] and Hu and Bentler [87]. Based on these indices, the data fits well with the measurement model.

### 4.5. Structural Equation Modeling (SEM)

In the current study, we utilized structural equation modeling to investigate the direct impact of GPQ on GS, GT, and CGBIs besides exploring the direct impact of GS and GT on CGBIs in addition to examining the potential effect of GS and GT as mediators in the nexus between GPQ and CGBIs. The results of the study’s structural model are presented in Table 5. Model fit measures indicate that the proposed structural model is well-fitted as recommended by Hair et al. [88] (see Table 5).

**Figure 2:**
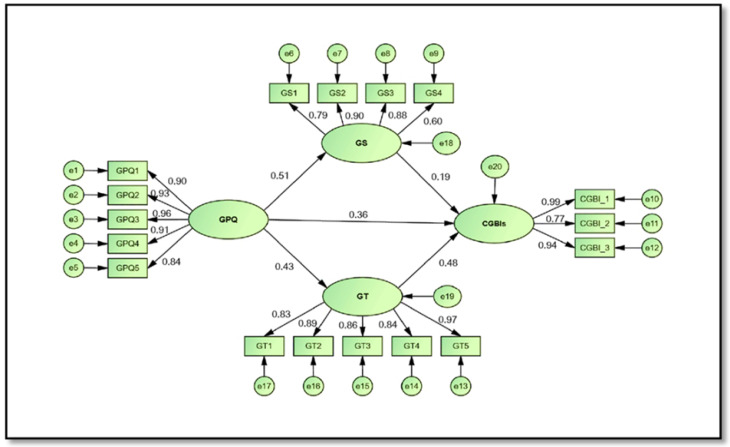
The structural model.

Regarding the direct and indirect nexuses among constructs of the study, the results presented in Figure 2 and Table 5 reveal that all the estimated paths were positive and significant, and all hypotheses were accepted. Hypothesis H_1_ which predicted that GPQ has a significant and positive effect on CGBIs is accepted (*β* = 0.364, *t*-value = 6.794, *p* < 0.001). Furthermore, H_2_ postulated that GPQ significantly positively affects GS is also supported (*β* = 0.511, *t*-value = 9.518, *p* < 0.001). In addition, GT is directly significantly affected by GPQ (*β* = 0.432, *t*-value = 8.034, *p* < 0.001). Hence, H_3_ is accepted. Additionally, the findings of the SEM supported H_4_ and H_5_ which assumed that GS and GT positively significantly impact CGBIs, respectively (*β* = 0.194, *t*-value = 3.621, *p* < 0.001; *β* = 0.481, *t*-value = 8.946, *p* < 0.001). 

In order to validate the potential mediating effect of GS and GT in the nexus between GPQ and CGBIs, a Bootstrapping technique was adopted. Table 5 laid emphasis on the significant positive impacts of GPO on CGBIs through GS (*β* = 0.099, *t*-value = 1.847, *p* < 0.05). As a result, H_6_ which predicted that GS has a significant mediating effect on the nexus between GPQ and CGBIs is accepted. Likewise, the findings of the bootstrapping approach exhibited that CGBIs are indirectly (via GT), significantly, and positively affected by GPQ (*β* = 0.208, *t*-value = 2.277, *p* < 0.05), confirming H_7_. In accordance with the recommendation of Kelloway [89] and Zhao et al. [90] for full and partial mediation, the indirect paths of the previous nexus (GPQ-CGBIs) were reviewed to examine the mediation effect of GS and GT. Their findings validated that full mediation can only be established when the direct effect is insignificant, but the indirect effect is significant. Meanwhile, partially mediating paths occur when both effects (direct and indirect) are significant. Accordingly, the results from SEM indicate that GS and GT significantly partially mediate the positive nexus between GPQ and CGBIs, in which both direct and indirect paths are significant.

## 5. Discussion

The current study primarily aims at empirically exploring the nexuses between green perceived quality (GPQ), green satisfaction (GS), green trust (GT), and customers’ green behavioral intentions (CGBIs) in a sample of five-star eco-friendly hotels in Egypt. The SEM with the bootstrapping technique was employed for analyzing the direct and indirect nexuses between GPQ, GS, GT, and CGBI. The findings of the study are discussed as follows:

Based on the results of SEM that ascertain the nexuses between the constructs of the study (GPQ, GS, GT, and CGBIs), we first concluded that GPQ significantly and positively affects CGBIs, meaning that the increase in GPQ not just only meets the customers’ environmental needs and desires but also plays an imperative role in enhancing their green behavioral intentions toward staying at green/eco-friendly hotels besides recommending them to others as well as paying a premium for hotel green products and services. Compared with the literature review, it could be noticed that these findings are consistent with those concluded by Wasaya et al. [72], Alexandris et al. [57], Yu et al. [58], and Riva et al. [25]. Their findings confirmed that GPQ is the key determinant of CGBIs. Unlike the findings of the previous studies, Assaker et al. [14] proved that hotel-perceived quality doesn’t directly significantly affect loyalty behavioral intention. Following the previous findings, it is possible to conclude that a higher GPQ increases the perception of CGBIs.

Second, the findings of the study also clarified that GS is significantly positively affected by GPQ, implying that the higher GPQ, the better the contribution to enhancing GS. This finding is consistent with previous studies, i.e., [1,29,60], which concluded that GPQ is the key predictor of GS. For instance, the results of the empirical study conducted by Jil and Jacob [29] assured that the increase in GPQ significantly improves green satisfaction (*β* = 0.539, *p* > 0.001). Similarly, the study conducted by Chen and Chang [1] concluded that GS is significantly impacted by GPQ. From the previous findings, it could be concluded that the greater the GPQ, the higher the satisfaction with eco-friendly hotels’ products and services. 

Third, the findings of the study reveal the positive direct impact of GPQ on GT, which confirms the findings of the existing research i.e., [1,29,66,67,68]. With these results, it could be advocated that GPQ plays a significant role in developing GT. It means that to trust a product/service, the customer must perceive the best quality of this product/service. In contrast to the previous findings, the empirical study conducted by Imaningsih [60], in the body shop product context, illustrated that GPQ positively but not significantly affects green trust (*β* = 0.026, *t* = 0.107, *p* = 0.915). From the previous findings, it could be concluded that the greater the GPQ, the better the GT. 

Fourth, the result of the study showed that CGBIs were found to be influenced by GS. The significance of the influence of GS on CGBIs has been emphasized by Chen et al. [27] and Al-Quran et al. [70]. The GS has been mentioned as a key predictor of CGBIs such as GWoM [32], customer repurchase intention of green products [71], customer retention and repurchase intention [33], and green customer loyalty [72]. From the previous findings, it is possible to assume that the greater the perceived GS, the greater the perceived CGBIs (i.e., intention to stay at green hotels—intention to positive WoM as well as Intention to pay a premium).

Fifth, regarding the nexus between GT and CGBIs, the study results confirm the significant role of GT in forming CGBIs. The findings validate that GT significantly and positively influences CGBIs. These findings are in agreement with the previous studies which assured that GT is a prerequisite for CGBIs. Trust was regarded as a vital factor affecting customers’ choice of green products/services [30]. GT contributes to showing positive GPI [1,73]. This finding supports Lam et al. [71] who concluded that GT has a positive and significant impact on customer repurchase intention of green products (*β* = 0.37, *t* = 6.60, *p* < 0.001). Further, this finding agreed with the finding of Sultana et al.’s study [91] which demonstrated that GT has a significant positive effect on customers’ intention to stay at green hotels in Bangladesh. Another study conducted by Guerreiro and Pacheco [74] concluded that GT is significantly correlated with GWoM and GPI. In their study, they argued that consumers are less likely to purchase or spread positive GWoM about a brand if they do not trust its green claims and/or actions.

Finally, concerning the mediating effect of GS and GT in the nexus between GPQ and CGBIs, the findings of this study show that both GS and GT have a partial mediation effect on the GPQ-CGBIs relationship. These findings are in line with those concluded by Jil and Jacob [29] who found that green purchasing intention is indirect, through GS and GT, influenced by GPQ. Further, in the non-green marketing contexts, Bou-Llusar et al. [61] and Konuk et al. [37] advocated that customer satisfaction has a significant partial mediation effect on the nexus between perceived quality and customer behavioral intention (i.e., intention to purchase and positive WoM). In terms of GT’s mediating role in the relationship between GPQ and CGBIs, the findings of this study support the results of Zulfanizy and Wahyono [8], indicating that GT has a significant role as an intervening variable in the relationship between GPQ and customers’ green purchase intention. Thus, it could be suggested that the higher the GPQ, the better the GS, which in turn contributes to promoting the CGBIs toward green hotels’ products and services. Similarly, the greater the GPQ, the greater the GT, which results in a significant increase in CGBIs to eco-friendly hotels.

## 6. Conclusions of the Study

The current study primarily aims at empirically exploring the nexuses between green perceived quality (GPQ), green satisfaction (GS), green trust (GT), and *customers*’ green behavioral intentions (CGBIs) in a sample of five-star eco-friendly hotels in Egypt. To achieve this aim, a self-administrated questionnaire was developed and distributed to a convenience sample of local guests staying at the certified five-green star hotels. The study findings *revealed* that GS, GT, and CGBIs are significantly positively affected by GPQ. Furthermore, GT and GS have a significant positive effect on CGBIs. Moreover, GT and GS *significantly* partially mediate the nexus between GPQ and CGBIs. From the previous findings, it could be concluded that the increase in investment for enhancing GPQ significantly contributes to the improvement in GS, GT, and CGBIs. Additionally, the higher the GT, GPQ, and GS, the greater the revisit intention to green hotels, positive green word-of-mouth (GWoM), and intention to pay a premium for staying in environmentally friendly hotels. Thanks to the findings of the study, some theoretical and practical implications along with further research could be suggested as follows.

### 6.1. Theoretical Implications

Several theoretical implications can be drawn from the findings of this study. Firstly, this research significantly contributes to the literature on CGBIs in the green hotel context by providing insights into the nexus between GPQ, GS, GT, and CGBIs. The study findings illustrated the significant positive direct and indirect (via GS and GT) interrelationship between GPQ, and CGBIs. Furthermore, the findings of the study establish the imperative contribution of GPQ as an independent variable in predicting GT, CGBIs, and GS. Secondly, in the hospitality industry context, GS and GT are regarded as key determinants of customers’ green behavioral intentions. Among the constructs examined, GT was the most effective predictor of CGBIs.

Thirdly, as far as the authors of this study know, no study has empirically examined GS and GT as intermediary variables in the nexus between GPQ and CGBIs in the green hotel industry context, particularly in the developing countries. In this study, the indirect relationship between GPQ and CGBIs was based on the S-O-R model. Using this model, the nexus between GPQ (stimulus), GS, and GT (organism-related factors), as well as CGBIs (response) was explored. Based on the findings of this study, GS and GT play a partial mediating role in the relationship between GPQ and CGBIs. As a result, the body of literature on GS and GT has been extended as GS and GT have been shown to act as significant mediators. Further, these findings support and extend the S-O-R model in the green hospitality industry context. It appears from the results of this study that higher GPQ significantly enhances customer satisfaction, sustains trust, and builds long-term relationships, consequently contributing to the greater revisit intention to green hotels, positive green word-of-mouth (GWoM), and intention to pay a premium for staying in environmentally friendly hotels. 

Fourthly, a new model indicating GPQ, GS, GT, and CGBI has been developed and validated, which contributes to deepening the green marketing literature related to the hospitality industry context. The findings of the study were significant since the developed model could be utilized as a guide for future research aims at boosting CGBI in the hospitality industry context. Fifthly, this study expands the research on perceived quality, trust, satisfaction, and consumer behavioral intention in the green hotel context. The findings of this study provide an in-depth understanding of the key factors positively affect CGBI which may be valuable insights for scholars in the field of hospitality research. 

### 6.2. Practical Implications

In terms of practical implications, several implications for green/eco-friendly hotel operators and governments should be considered, especially in the context of the developing countries. First, the study findings reveal that GPQ contributes significantly to improving CGBIs in three ways. The first way is that GPQ directly positively affects CGBIs. The second way is that CGBIs are significantly enhanced, indirectly via GS, by GPQ. The third one is that GPQ has a significant positive impact on CGBIs indirectly through GT. Hence, green hotel operators should give significant attention to enhancing the quality of green products and services provided. For this purpose, the quality of hotels’ products and services should be reliable and durable concerning environmental consideration and performance. 

Second, the study’s findings prove that GPQ is the key predictor of GS and GT. As a result, hotel operators must encourage better investment in improving the quality of products and services offered not only to enhance customers’ GS but also to maintain GT besides developing long-term relationships with their customers. Third, in the long run, due to their significant impacts on promoting CGBIs such as the intention to revisit/ stay at eco-friendly hotels, spread positive WoM, and pay more for staying in environmentally friendly hotels, hotel operators should consider GPQ, GS, and GT into their strategical environmental plans. Fourth, GT as well as GS have significant positive impacts on CGBIs. Upon that, hotel operators/managers should be keen to pay careful attention towards increasing GS among the hotels’ customers, being reliable, dependable, and trustworthy in terms of their environmental performance, and keeping their promises related to environmental improvements. 

Fifth, the results of the study showed that GT and GS play a significant mediation role between GPQ and CGBIs. Upon that, building GT and increasing GS among hotel customers, to enhance the extent of the positive nexus between GPQ and CGBIs, is essential. Sixth, measuring customers’ satisfaction with green products frequently is very crucial. To improve green behavior intentions, it is imperative to consider customers’ feedback and comments regarding the quality of green products and services provided. Finally, with the growing demand for green products and services in the hospitality industry context, government and public authorities should take the lead in promoting green investment in the hotel business by developing policies that encourage, support, and promote its implementation. These policies may include lowering taxes paid, guaranteeing credits as well as providing grants for green technology purchases.

### 6.3. Limitations and Further Research

There are several limitations to the current study. Firstly, our study applied GPQ, GS, GT, and CGBIs only to five-star eco-friendly hotels in Egypt focusing on local guests. Findings may not be generalizable to other nations or populations. It would be helpful to conduct further research using a larger sample size to gain more insight. Secondly, a cross-cultural study could also be conducted in future research to ascertain cultural differences. Thirdly, this limitation is relevant to the data collection method, where the study participants responded to a self-administered questionnaire based on their subjective perspectives. It may be possible to gain a deeper understanding with a mixed-method approach (qualitative and quantitative). Fourthly, the study explored the possible mediation effect of GS and GT on the nexus between GOQ and CGBIs. Future research might examine other variables, such as green image and green perceived value, as potential mediators. Fifthly, other moderator variables, such as tourists’ pro-environmental awareness, environmental values, environmental concerns, and environmental friendliness, may also be considered. Sixthly, the study examined CGBIs in a one-dimensional concept. Therefore, further research on CGBIs could be conducted in multiple dimensions (i.e., green word-of-mouth, green purchase intention, intention to pay a premium for green products…etc.) separately.

## Figures and Tables

**Table 1:** Respondents’ demographic characteristics.

Characteristic	No.	%
Gender		
Male	138	45.4
Female	166	54.6
Age		
18 to less than 30 years	97	31.9
30 to 40 years	110	36.2
41 to 50 years	74	24.3
More than 50 years	23	7.6
Educational level		
High school degree or equivalent	32	10.5
University degree	185	60.9
Postgraduate degree	87	28.6
Monthly income		
Less than $2000	45	14.8
$2000 to $3000	115	37.8
$3001 to $4000	77	25.3
More than $4000	67	22.1
Marital status		
Single	118	38.8
Married	166	54.6
Other (widowed, divorced)	20	6.6
Total	304	100%

**Table 2:** Descriptive statistics, Reliability, and Confirmatory Factor Analysis Properties.

Construct	Items	M (S.D.)	Std. Loading (CFA) ^1^	Cronbach’s Alpha	CR^2^	AVE^3^
Green Perceived Quality (GPQ)	GPQ1	4.42 (0.90)	0.901 ***	0.959	0.959	0.824
	GPQ2	4.48 (0.86)	0.927 ***			
	GPQ3	4.47 (0.86)	0.960 ***			
	GPQ4	4.35 (0.88)	0.910 ***			
	GPQ5	4.31 (0.86)	0.838 ***			
Green Satisfaction (GS)	GS1	4.30 (0.80)	0.787 ***	0.863	0.874	0.639
	GS2	4.30 (0.83)	0.899 ***			
	GS3	4.35 (0.83)	0.879 ***			
	GS4	4.24 (0.90)	0.598 ***			
Green Trust (GT)	GT1	4.35 (0.97)	0.828 ***	0.944	0.944	0.770
	GT2	4.30 (1.02)	0.892 ***			
	GT3	4.27 (0.99)	0.859 ***			
	GT4	4.17 (0.99)	0.837 ***			
	GT5	4.19 (1.07)	0.965 ***			
Customers’ Green Behavioral Intentions (CGBIs)	CGBI_1	4.22 (0.96)	0.965 ***	0.927	0.933	0.825
	CGBI_2	4.21 (0.97)	0.994 ***			
	CGBI_3	4.17 (0.94)	0.774 ***			

M = mean, S.D. = Standard deviation, Std. Loading, (CFA) ^1^ = Standardized Factor Loading, CR^2^ = Composite Reliability, AVE^3^ = Average Variance Extracted, *** *p* < 0.001.

**Table 3:** Discriminant Validity Based on the Fornell–Larcker Criterion.

Construct	GPQ	GS	GT	CGBIs
1- Green Perceived Quality (GPQ)	0.908 ^a^			
2- Green Satisfaction (GS)	0.532 *** ^b^	0.800 ^a^		
3- Green Trust (GT)	0.453 *** ^b^	0.282 *** ^b^	0.877 ^a^	
4- Customers’ Green Behavioral Intentions (CGBIs)	0.429 *** ^b^	0.211 *** ^b^	0.521 *** ^b^	0.908 ^a^

Note: ^a^ AVE’s square root, *** ^b^ latent variables correlation (*** *p* < 0.001).

**Table 4:** Discriminant Validity via HTMT.

Construct	GPQ	GS	GT	CGBIs
1- Green Perceived Quality (GPQ)				
2- Green Satisfaction (GS)	0.554			
3- Green Trust (GT)	0.457	0.297		
4- Customers’ Green Behavioral Intention (CGBIs)	0.456	0.217	0.571	

Note: The HTMT should not exceed 0.85.

**Table 5:** Structural Parameter Estimates.

Hypothesized Path					Standardized Path Coefficients	*t*-Value	Results
Direct Path							
**H_1_**: GPQ	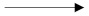	CGBIs			0.364	6.794 ***	Accepted
**H_2_**: GPQ	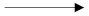	GS			0.511	9.518 ***	Accepted
**H_3_**: GPQ	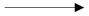	GT			0.432	8.034 ***	Accepted
**H_4_**: GS	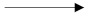	CGBIs			0.194	3.621 ***	Accepted
**H_5_**: GT	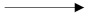	CGBIs			0.481	8.946 ***	Accepted
Indirect path							
**H_6_**: GPQ	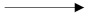	GS	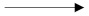	CGBIs	0.099	1.847 *	Accepted
**H_7_**: GPQ	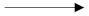	GT	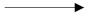	CGBIs	0.208	3.882 ***	Accepted

Note: GPQ = green perceived quality, GS = green satisfaction, GT = green trust, CGBIs = customers’ green behavioral intentions, model fit: x2/df = 3.112 *** *p* < 0.001, RMR = 0.042, RMSEA = 0.068, GFI = 0.908, IFI = 0.958, NFI = 0.948, RFI = 0.928, CFI = 0.961, *** p < 0.001, * p < 0.05.

## Data Availability

The data presented in this study are available on request from the corresponding authors.

## Appendix A

Study’s constructs and their related items.

**Construct**	**Items**	**Statement**
Green Perceived Quality (GPQ)	GPQ1	In terms of environmental concerns, the quality of the hotel’s products/services is perceived to be the best benchmark.
	GPQ2	The hotel’s products/services quality is reliable in terms of environmental requirements.
	GPQ3	The hotel’s products/services quality is durable in terms of environmental performance.
	GPQ4	The hotel has an excellent environmental image because of the quality of its product and services.
	GPQ5	In terms of environmental reputation, the hotel’s products/services have professional quality.
Green Satisfaction (GS)	GS1	Because of its environmental image, you are happy that you select to stay at this hotel
	GS2	Due to the hotel’s environmental functionality, you decide to purchase its products/services.
	GS3	Generally, you are pleased with the hotel’s eco-friendly products/services.
	GS4	Your overall satisfaction with this hotel can be attributed to its environmental performance.
Green Trust (GT)	GT1	You think that the hotel’s environmental image is generally regarded as reliable.
	GT2	Generally, you believe that the hotel’s environmental function is dependable.
	GT3	In general, you think that claims made about the hotel’s environmental impacts are trustworthy.
	GT4	The environmental performance of this hotel meets your expectations.
	GT5	The hotel is keeping its promises regarding environmental improvement.
Customer Green Behavioral Intentions (CGBIs)	CGBI_1	I intend to stay at eco-friendly hotels.
	CGBI_2	Often, I recommend eco-friendly hotels to my family and colleagues.
	CGBI_3	I intend to pay more for an eco-friendly hotel is acceptable.
